# Awareness and knowledge about prostate cancer among male teachers in the Sunyani Municipality, Ghana

**DOI:** 10.4314/ahs.v21i2.22

**Published:** 2021-06

**Authors:** Bernard Yeboah-Asiamah Asare, Mercy Mawufenya Ackumey

**Affiliations:** 1 Department of Environmental Health and Sanitation, Faculty of Science and Environmental Education, University of Education, Winneba, Asante-Mampong Campus, Ghana; asiamahyeboah2006@yahoo.com; 2 Department of Social and Behavioural Sciences, School of Public Health, University of Ghana, Ghana; jekammy@yahoo.com

**Keywords:** prostate cancer, awareness, knowledge, male teachers, Ghana

## Abstract

**Objective:**

The study was aimed at assessing the awareness and knowledge of prostate cancer (PC) among male teachers in the Sunyani municipality of Ghana.

**Methods:**

This was a cross-sectional study conducted using a structured questionnaire to collect data from 160 male teachers aged 45 years or more, randomly selected from public elementary and high schools in the Sunyani Municipality. Pearson's Chi square and Fishers exact tests were used to examine the association between socio-demographic characteristics and knowledge of PC.

**Results:**

On average, respondents were aged 50±3.95 years. There was a universal awareness of PC. Most of the respondents could identify at least one signs and symptoms of PC (88.1%), risk factors of PC (78.8%), and indicated that PC could be treated through surgery (70.6 %), but only 37.5% of respondents knew about screening tests for PC. The study found 57.5% of them had adequate knowledge about PC. Socio-demographics characteristics were not associated with knowledge about PC. Main sources of information were the television (68%) and radio (57 %).

**Conclusion:**

The outcomes of the study suggest the need for general educational campaigns with emphasis on modalities for the screening of PC using the appropriate media channels for accessibility.

## Introduction

Prostate cancer (PC) has over the years emerged as a public health concern,^1^,^2^ as it has become the second common cancer and the fifth leading cause of death among men worldwide.^3^ Globally, 1.3 million new cases and 359, 000 related deaths were projected in 2018.^3^ PC incidence are the highest among American men of Caucasian and African origin^2^,^4^ and account for most of the cancer related-deaths among men in sub-Saharan Africa.^3^ In 2015, about 25,000 cases of PC were reported in Africa.^5^

Early screening and detection of PC is one of the best ways of reducing PC related deaths.^2^,^6^ Yet, PC cases are reported in the late stages of the disease,^1^,^7^ mostly due to lack of awareness, inadequate knowledge about the disease, unavailability of screening facilities,^8^–^10^ absence of adequate educational programs and interventions which are known to increase awareness and PC knowledge levels.^9^,^11^–^13^ Inadequate knowledge about PC has widely been identified. An Australian study among civilian men and men of the Australian Defence Force reported low levels of knowledge on symptoms, screening and treatment in both groups.^14^ Studies conducted among African-American, Black Caribbean and Hispanic Americans,^8^,^9^,^13^,^15^ adult Ugandan men residing in Kampala^16^ and Nigerian men visiting a hospital in Lagos^17^ have also reported low levels of PC knowledge. There are inconclusive views on the factors that influence men's knowledge of PC. Some studies argue that high knowledge levels of PC are related to high education and income levels.^11^,^13^,^18^,^19^ Evidence from other studies have also suggested that men at risk of PC, particularly those with family history of PC^20^,^21^ and men previously diagnosed of PC^8^ have adequate knowledge of PC and screening, yet fail to have the screening done. The Ghana Health Service reports a lack of PC awareness among the general population,^22^ although late stage PC account for three-fourths of cases seen at health facilities.^22^–^24^ In Africa, awareness of and early detection is generally not common^25^ with a reported prevalence of PC screening of around 2.5%.^24^ Men reporting with late stages of PC at health facilities in Ghana may be ascribed to ignorance and/or not been well informed about the disease.^22^ Raising general awareness about PC has been underscored as significant particularly in developing countries.^25^

Professional workers are regarded as informed individuals as such expected to be knowledgeable about several issues including health, however it has been established in sub-Saharan Africa that professional workers even including health workers are inadequately knowledgeable about several related health issues, and have been associated with poor health screening.^26^ For instance, Arulogun and Maxwell have reported an average knowledge about cervical cancer and poor screening for cervical cancer among professional nurses in Nigeria.^26^ Teachers are agents of change in the society,^27^ and as such could be important target in health programs aimed at the promotion of the screening of PC. However there is paucity of research on PC cancer in Ghana particularly concerning awareness and knowledge of PC among such population, which is an important prerequisite for public health programming for PC education and screening. This study examined the awareness and knowledge of prostate cancer among male teachers in the Sunyani municipality of Ghana.

## Methods

### Study design and setting

The study used a cross-sectional design and was done as part of a bigger study on knowledge, perceptions and attitudes towards PC among teachers in the Sunyani Municipality, in the Bono Region of Ghana between May and July 2015. Sunyani Municipality has an estimated population of 147,301 as at 2010 with 49.6% of them being males. The Municipality has 59 primary and 54 junior high public schools, 4 public second cycles Schools and 3 tertiary institutions. There are also several numbers of private schools. The Sunyani Municipality host the regional hospital, and has 4 other hospitals, 14 clinics, and 8 health centres. The regional hospital offers a screening and treatment services for PC, and there are several private medical laboratories which offer PC screening services.

### Study population, Sample size and sampling procedure

The study was conducted among male teachers in the Sunyani Municipality, and recruited those aged 45 years or more from public schools into the study. This study population was selected because men in this year group are potentially at risk of developing PC. Also the Ghana Health Service recommends that early screening for PC should start for men at 40 years^22^. This suggests that men of 45 years and above are expected to have been screened for at least twice for PC. The public schools were chosen as the focus because teachers in these schools are documented. The Municipality has a total of 1276 teachers of which 625 were males; and 256 out of the 625 were aged 45 years or more (as at September, 2014) (Sunyani Municipal Education Service). Using the Krejcie and Morgan table, 1970, 28 156 (rounded up to 160) male teachers were estimated to be recruited. The calculation was based on a population of 256 male teachers 45 years and above, an age range considered high risk for PC.

### Sampling method

The schools within the Sunyani Municipality were grouped into 3 different strata; Primary, Junior High School (JHS), and Senior High School (SHS). The proportion of teachers to be sampled from each strata was computed by dividing the number of male teachers aged 45 and above in the strata by the total number of teachers aged 45 years and above in the municipality. The result was multiplied by the computed sample size of 156 (Table 1).

**Table 1:** Computation of proportion of teachers to be sampled from each stratum

School type	Number of male teachers aged 45 and above	Number of teachers sampled
Primary	59	59÷256*156=36
JHS	107	107÷256*156=65
SHS	90	90÷256*156=55
Total	256	156

A list of schools was obtained from the Sunyani Municipal Education Office. The schools were grouped into 3 strata – Primary, Junior High School and Senior High School. The number of schools sampled from each stratum was computed by dividing the number of teachers sampled for each stratum by the number of male teachers aged 45 years and above for each stratum. The result was multiplied by the total number of schools in each stratum (Table 2). A simple random sample technique was then used to select the required schools from the list. Using a simple random sampling technique the required number of schools for each stratum was selected. Subsequently teachers were randomly selected and interviewed.

**Table 2:** Computation of sampled schools for each stratum

Stratum (Schools)	Number of male teachers aged 45 and above	Number of teachers sampled	Number of schools in the municipality	Number of schools sampled
Primary schools	59	59÷256*156=36	59	36÷59*59=36
JHS	107	107÷256*156=65	54	65÷107*54=33
SHS	90	90÷256*156=55	4	55÷90*4=2

### Data collection tool and data analysis

Authors formulated a structured questionnaire to collect data on respondents' demographics, knowledge and sources of information about PC. The questionnaire was pre-tested in the Sunyani West district and revised to make the necessary corrections after pre-testing. Questionnaire was designed and face-to-face interviews conducted in English by the authors. Interviews were conducted in the schools respecting the privacy of the respondents, and lasted for an average of 10 minutes per respondent.

Data were entered, cleaned and analysed using STATA 12 (StataCorp LP, College Station, TX, USA). Data on background characteristics, knowledge and sources of information on PC were summarized using frequencies and proportions for descriptive purposes. Awareness of PC was defined as whether the respondent had ever heard about PC. Knowledge of PC was assessed on causes, signs and symptoms, and treatment of PC with the responses “agree”, “don't know” and “don't agree”; scored and classified into low and high knowledge as detailed elsewhere.^29^ The relationships between background characteristics and knowledge of PC were tested using where applicable the Pearson's Chi square (χ2) and Fishers exact test. P<0.05 was considered significant for all statistical procedures.

### Ethics approval and consent to participate

The study was approved by the Ghana Health Service's Ethical Review Board (GHS-ERC: 110/02/15). Voluntary and written informed consent was obtained from all participants.

## Results

### Background characteristics of respondents

One hundred and sixty respondents with an average age was 50±3.95 years took part in the study. Majority of them were married (87.5%), and had obtained a University degree (90.6%). Half of the respondents (50%) were ranked as Assistant Directors in the Ghana Education Service (Table 3).

**Table 3:** Distribution of socio-demographic characteristics by knowledge about prostate cancer

Characteristics	Freq. (%)	Knowledge level, n(%)		*X^2^(df)*	*p*-value
				
		Low	High		
**Age**					
45–50	109 (68.1)	45(66.2)	64(69.6)		
51–55	34 (21.3)	14(20.6)	20(21.7)	0.8487(2)	*0.654
56–60	17 (10.6)	9(13.2)	8(8.7)		
**Marital status**					
Single	20(12.5)	9(13.2)	11(12.0)		
Married	140(87.5)	59(86.8)	81(88.0)	0.0585(1)	*0.809
**Educational status**					
College of education	15(9.4)	60(88.2)	85(92.4)		
University graduate	145(90.6)	8(11.8)	7(7.6)	0.7949(1)	*0.373
**Current rank**					
Senior	14(8.6)	7(10.3)	7(7.6)		
Superintendent					
Principal	66(41.4)	30(44.1)	36(39.1)		
Superintendent					
Assistant Director II	63(39.4)	27(39.7)	36(39.1)		**0.386
Assistant Director I	17(10.6)	4(5.9)	13(14.1)		
**Religious affiliation**					
Christian	145(90.6)	60(88.2)	85(92.4)		
Muslim	15(9.4)	8(11.8)	7(7.6)	0.7949(1)	*0.373
**Ethnicity**					
Akan	105(65.8)	44(64.7)	61(66.3)		
Ga	3(1.9)	3(4.4)	0(0.0)		
Ewe	18(11.3)	7(10.3)	11(12.0)		**0.419
Northern	28(17.5)	12(17.7)	16(17.4)		
Others	6(3.8)	2(2.9)	4(4.4)		

Data are presented in frequency (n) and percentages (%); *X*^2^= chi-square statistic; *df*=degree of freedom

* *P*-value from chi-square test

** *P*-value from fisher's exact test

### Awareness of PC and sources of information

All 160 (100%) respondents had heard about PC. Sources of information for PC included television (68.8%), radio (57.5%), newspapers (21.3%) and health professionals (20.0 %) (Table 4).

**Table 4:** Awareness of prostate cancer

Parameter	Frequency	Percent
**Ever heard of prostate** **cancer**	160	100
**Sources of information** *		
Television	110	68.8
Radio	92	57.5
Newspaper	34	21.3
Health pamphlet	8	5.0
Church/Mosque	15	9.4
Internet	13	8.1
Books/journals	9	5.6
Health professionals	32	20.0
Family and friends	18	11.3

* multiple respondents allowed

### Knowledge about PC

Table 5 shows the distribution of respondent's knowledge about PC. Most of the respondents (63.1%), indicated PC has a known cause and 37.5% of respondents mentioned PC may not present with signs and symptoms at the early stages of the disease. Most of the respondents (88.1%) could identify at least one signs and symptoms of PC; indicating difficulty urinating (80%), weakness and numbness in the legs and feet (35%), bloody urine (45 %) and pain in the waist and back (41.9%). Also, about 79% of the respondents indicated at least one risk factor of PC; indicating previous history of PC in the family (51.9%), and men aged above 40 years (42.5%). Concerning knowledge about screening tests and treatment options for PC, only 37.5% of respondents indicated Prostate Specific Antigen (PSA) blood test and Digital Rectal Examination (DRE) were screening tests for PC while most respondents indicated that PC could be treated through surgery (70.6 %) (Table 5). The study found only 57.5% of respondents had adequate knowledge about PC. Chi-square and Fisher's exact tests both showed no were statistical significant relationship between socio-demographic characteristics and knowledge about PC (p>0.05) (Table 3).

**Table 5:** Distribution of respondent's knowledge levels about PC (N=160)

Statements on knowledge	Agree, n (%)	Don't know, n (%)	Don't Agree, n (%)
*Prostate cancer has no known cause*	32 (20.0)	27 (16.9)	101 (63.1)
*Prostate cancer may not present with* *signs and symptoms at the early stages*	60 (37.5)	28 (17.5)	72 (45.0)
*Difficulty in urinating may be a warning* *sign of PC*	128(80.0)	18(11.2)	14(8.8)
*Weakness and numbness in the leg and* *feet may be a warning sign of PC*	56(35.0)	80(50.0)	24(15.0)
*Passing bloody urine may be warning* *sign of prostate cancer*	72(45.0)	39(24.4)	49(30.6)
*Prostate cancer may present with pain in* *the waist and back*	67(41.9)	66(41.2)	27(16.9)
*Men aged 40 and below are not at risk of* *developing prostate cancer than older* *men*	68(42.5)	24(15.0)	68(42.5)
*Men who have a previous history of* *prostate cancer in the family are at risk* *of PC*	83(51.9)	31(19.4)	46(28.7)
*Prostate Specific Antigen (PSA) and* *Digital Rectal Examination (DRE) are* *screening methods for PC*	60(37.5)	100(62.5)	0(0.0)
*Prostate cancer can be treated through* *surgery*	113(70.6)	37(23.1)	10(6.3)

## Discussion

This study was undertaken to assess knowledge and the sources of information about PC among male teachers in the Sunyani Municipality of Ghana. The study findings suggest a high level of awareness and knowledge of PC among study respondents. Knowledge on PC was not associated with socio-demographic characteristics of the teachers'.

Similar high levels of PC awareness have been reported among male University students in Ghana^29^ and staff in Nigeria,^30^ and public servants in Nigeria.^31^ Findings from this study were however, in contrast to findings from another study in Nigeria which established more than 90% of public servants were totally unaware of PC.^10^ This variation could be due to low educational levels among respondents in the previous study; as 23% of them had no education compared to all respondents in our study with high educational level. For it has been stipulated that advanced educational status and increase access to information could account for increase awareness and knowledge of PC.^31^

Even though the overall level of awareness of PC was high among study participants, only 57.5% of them had adequate knowledge about PC. However, knowledge on the signs, symptoms of PC and screening test for PC was relatively low. Many respondents did not know that PC has no known cause and may be asymptomatic in the early stages of the disease. Furthermore, many respondents were unaware of the Prostate Specific Antigen (PSA) blood test and Digital Rectal Examination (DRE) screening methods for PC. Our study findings compare favourably to findings of a study conducted in Nigeria, where most of the men in the study had completed tertiary education and showed high awareness of PC yet, knowledge of symptoms and risk factors was low.^15^ Similar observations have been made among public servants in Nigeria,^10^ the general public in Burkina Faso^32^ and Ugandan men.^18^ This suggests the need for more aggressive educational campaigns.

Understanding men's awareness and knowledge of PC is critical to public health programming for PC education and screening. Earlier studies present conflicting findings on factors that influence awareness and knowledge of PC. Socio-demographic characteristics such as education, age and marital status in the current study, had no significant association with PC, probably because frequencies of these variables were positively skewed. In a study among South African PC patients attending a urological clinic, age and marital status were not associated with knowledge of PC. However in the same study, level of education, race and language were found to influence knowledge about PC which is contrary to the findings of this study.^33^

The television and radio were the main sources of information on PC in this study. Similar findings were found in studies among South Africa men visiting a urological clinic^33^ and Filipino men in the United State.^34^ In contrast, a study among men from 3 Arab countries, Saudi Arabia, Egypt and Jordan, has documented physicians as the main source of information on PC.^35^ In Ghana most households have access to television and radio,^36^ and these media have been used in several health campaigns to create awareness on several health issues in Ghana.^37^,^38^ The proliferation of health information on the media in Ghana form part of Ghana's strategy to reducing morbidities and mortality from PC.^22^ Awareness creation and education of PC aims to increase knowledge of signs, symptoms and risk factors of PC to encourage screening to reduce PC-related morbidity and mortality. For desired results and better impacts, awareness campaigns should also advertise the benefits of PC screening and health centres where PC screening is done.

The limitation of the study is acknowledged. Findings from the study were based on data collected from only teachers of public schools in the Sunyani Municipality; and as such limit its generality to all teachers and general populace in the municipality.

## Conclusion

The study has shown high level of awareness and knowledge about PC, but limited knowledge on the screening modalities for PC. Knowledge on PC was not associated with the socio-demographic characteristics of teachers. The outcomes of the study suggest the need for educational campaigns with emphasis on modalities for the screening of PC using the appropriate media channels for accessibility, to continuously increase awareness about PC and its screening.

## References

[1] Udeh EI, Amu OC, Nnabugwu II, Ozoemena O (2015). Transperineal versus transrectal prostate biopsy: our findings in a tertiary health institution. Niger J Clin Pract.

[2] Haas GP, Delongchamps N, Brawley OW, Wang CY, de la Roza G (2008). The worldwide epidemiology of prostate cancer: perspectives from autopsy studies. Can J Urol.

[3] Bray F, Ferley J, Soerjomataram I, Siegel RL, Torre LA, Jemal A (2018). Global cancer statistics 2018: GLOBOCAN estimates of incidence and mortality worldwide for 36 cncers in 185 countries. Cncer J Clin.

[4] Rebbeck TR, Devesa SS, Chang BL, Bunker CH, Chengl I, Cooney K (2013). Global patterns of prostate cancer incidence, aggressiveness, and mortality in men of african descent. Prostate Cancer.

[5] Adeloye D, David RA, Aderemi AV, Iseolorunkanmi A, Oyedokun A, Iweala EEJ (2016). An estimate of the incidence of prostate cancer in Africa: a systematic review ana meta-analysis. PLoS One.

[6] Cuzick J, Thorat MA, Andriole G, Brawley OW, Brown PH, Culig Z (2014). Prevention and early detection of prostate cancer. Lancet Oncol.

[7] Ali M (2011). Clinical presentation, pathological pattern and treatment options of prostate cancer at Al-Azhar University Hospitals over the last 30 years. Afr J Urol.

[8] Allen JD, Kennedy M, Wilson-Glover A, Gilligan TD (2007). African-American men's perceptions about prostate cancer: implications for designing educational interventions. Soc Sci Med.

[9] Chan EC, McFall SL, Byrd TL, Mullen DP, Volk RJ, Ureda J (2011). A community-based intervention to promote informed decision making for prostate cancer screening among Hispanic American men changed knowledge and role preferences: a cluster RCT. Patient Educ Couns.

[10] Ajape AA, Babata A, Abiola OO (2010). Knowledge of prostate cancer screening among native African urban population in Nigeria. Nig Q J Hosp Med.

[11] Winterich JA, Grzywacz JG, Quandt SA, Clark PE, Miller DP, Acuña J (2009). Men's knowledge and beliefs about prostate cancer: education, race, and screening status. Ethn Dis.

[12] Rajbabu K, Chandrasekera S, Zhu G, Dezylva S, Grunfeld EA, Muir GH (2007). Racial origin is associated with poor awareness of prostate cancer in UK men, but can be increased by simple information. Prostate Cancer Prostatic Dis.

[13] Wilkinson S, List M, Sinner M, Dai L, Chodak G (2003). Educating African-American men about prostate cancer: impact on awareness and knowledge. Urology.

[14] Sanderson R, Wijesinha SS, Jones KM (2013). What men know about the symptoms and treatment of prostate cancer: a study comparing ADF and civilian men. J Mil Veterans Health.

[15] Pedersen VH, Armes J, Ream E (2012). Perceptions of prostate cancer in Black African and Black Caribbean men: a systematic review of the literature. Psychooncology.

[16] Nakandi H, Kirabo M, Semugabo C, Kittengo A, Kitayimbwa P, Kalungi S (2013). Knowledge, attitudes and practices of Ugandan men regarding prostate cancer. Afr J Urol.

[17] Ogundele SO, Ikuerowo SO (2015). A Survey of the awareness of Prostate Cancer and its Screening among Men Attending the Outpatient Clinics of a Tertiary Health Center in Lagos, Nigeria. Niger J Surg.

[18] Deibert CM, Maliski S, Kwan L, Fink A, Connor SE, Litwin MS (2007). Prostate cancer knowledge among low income minority men. J Urol.

[19] Agbugui JO, Obarisiagbon EO, Nwajei CO, Osaigbovo EO, Okolo JC, Akinyele AO (2013). Awareness and knowledge of prostate cancer among men in Benin city, Nigeria. JMBR.

[20] Cormier L, Kwan L, Reid K, Litwin MS (2002). Knowledge and beliefs among brothers and sons of men with prostate cancer. Urology.

[21] Hevey D, Pertl M, Thomas K, Maher L, Chuinneagain SN, Craig A (2009). The relationship between prostate cancer knowledge and beliefs and intentions to attend PSA screening among at-risk men. Patient Educ Couns.

[22] Ministry of Health (2011). National Strategy for Cancer Control in Ghana, 2012 – 2016.

[23] Yamoah K, Beecham K, Hegarty SE, Hyslop T, Showalter T, Yarney J (2013). Early results of prostate cancer radiation therapy: an analysis with emphasis on research strategies to improve treatment delivery and outcomes. BMC Cancer.

[24] Chu LW, Ritchey J, Devesa SS, Quraishi SM, Zhang H, Hsing AW (2011). Prostate cancer incidence rates in Africa. Prostate Cancer.

[25] Taitt HE (2018). Global trends and prostate cancer: a review of incidence, detection, and mortality as influenced by race, ethnicity, and geographic location. Am J Men Health.

[26] Arulogun OS, Maxwell OO (2012). Perception and utilization of cervical cancer screening services among female nurses. Pan African Med J.

[27] Bourn D (2015). Teachers as agents of social change. IJDEGL.

[28] Krejcie RV, Morgan DV (1970). Determining Sample Size for Research Activities Educational and Psychological Measurement.

[29] Yeboah-Asiamah B, Yirenya-Tawiah D, Baafi D, Ackumey MM (2017). Perceptions and knowledge about prostate cancer and attitudes towards prostate cancer screening among male teachers in the Sunyani Municipality, Ghana. Afri J Urol.

[30] Binka C, Harrenson N, Doku DT, Antwi KAA (2014). Prostate Cancer Knowledge, Perceptions and Screening Behaviour among Male University Students in Ghana. IJSBAR.

[31] Ebuehi OM, Otumu IU (2011). Prostate Screening Practices Among Male Staff of the University of Lagos, Lagos, Nigeria. Afri J Urol.

[32] Oranusi CK, Mbieri UT, Oranusi IO, Nwofora AME (2012). Prostate cancer awareness and screening among male public servants in Anambra State, Nigeria. Afri J Urol.

[33] Kabore FA, Kambou T, Zango B, Ouedraogo A (2014). Knowledge and awareness of prostate cancer among the general public in Burkina Faso. J Cancer Educ.

[34] Mofolo N, Betshu O, Kenna O, Koroma S, Lebeko T, Claassen FM (2015). Knowledge of prostate cancer among males attending a urology clinic, a South African study. Springerplus.

[35] Conde FA, Landier W, Ishida D, Bell R, Cuaresma CF, Misola J (2011). Barriers and facilitators of prostate cancer screening among Filipino men in Hawaii. Oncol Nurs Forum.

[36] Arafa MA, Rabah DM, Wahdan IH (2012). Awareness of general public towards cancer prostate and screening practice in Arabic communities: a comparative multi-center study. Asian Pac J Cancer Prev.

[37] Ghana Satistical Service (2014). Ghana Living Standard Survey Round 6 (GLSS6): Poverty Profile in Ghana (2005–2013).

[38] Hill Z, Kirkwood B, Kendall C, Adjei E, Arthur P, Agyemang CT (2007). Factors that affect the adoption and maintenance of weekly vitamin A supplementation among women in Ghana. Public Health Nutr.

[39] Oppong Asante K, Oti-Boadi M (2013). HIV/AIDS knowledge among undergraduate university students: implications for health education programs in Ghana. Afri Health Sci.

